# Efficiency of Combining Heated Eye Mask with Intense Pulsed Light Therapy as a Treatment Option for Evaporative Dry Eye Disease

**DOI:** 10.22336/rjo.2024.29

**Published:** 2024

**Authors:** Cristina-Patricia Pac, Francis Ferrari, Nadina Mercea, Mihnea Munteanu

**Affiliations:** * Department of Ophthalmology, “Victor Babeş” University of Medicine and Pharmacy, Timişoara, Romania; ** Clinique Espace Nouvelle Vision, Paris, France; *** Department of Ophthalmology, Municipal Clinical Emergency Hospital, Timişoara, Romania

**Keywords:** dry eye disease, IPL therapy, heated eye mask, non-invasive first break-up time (NIFBUT), non-invasive average break-up time (NIABUT), ocular surface inflammatory evaluation (OSIE), tear film stability evaluation (TFSE), eye fitness test (EFT), central tear meniscus height (CTMH), thinnest tear meniscus height (TTMH)

## Abstract

**Background and objectives:** The study aimed to establish the efficiency of combining the Posiforlid heated eye mask with intense pulsed light therapy (IPL), as a treatment strategy for evaporative dry eye disease.

**Materials and methods:** This study included 110 patients, respectively 220 eyes, diagnosed with evaporative dry eye disease, patients between 18 and 86 years old, divided into two study groups. The first one, the control group, consisted of 73 patients treated with IPL therapy, and the second of 37 patients, who underwent IPL therapy associated with Posiforlid heated eye mask. Subjective evolution was assessed using an eye fitness test (EFT) regarding symptomatology. Objective assessment of the ocular surface was performed by tear film stability evaluation (TFSE), non-invasive first break-up time (NIFBUT), non-invasive average breakup time (NIABUT), ocular surface inflammatory evaluation (OSIE), measuring of the central tear meniscus height (CTMH) and thinnest tear meniscus height (TTMH). The assessment was performed at the beginning of the IPL treatment, during the IPL sessions, at the end of the IPL treatment, and afterward, at 3, 6, and 12 months.

**Results:** Tear film stability has increased in both study cases, but no statistically significant difference was observed between the two groups studied. For the control group, tear film stability evaluation (TFSE) started from 310.56 ± 389.54 at baseline (time 1 presentation) to 114.40 ± 122.90 after 12 months, and for the heated mask group, from 391.11 ± 456.45 (time 1 presentation) to 97.38 ± 105.98 after 12 months. NIABUT increased from 10.72 ± 4.90 seconds to 14.79 ± 3.72 seconds in the control group, and from 11.11 ± 5.08 seconds to 15.84 ± 2.26 seconds in the second group. OSIE decreased, as expected, from 7.18 ± 7.93 percent in the control group to 2.24 ± 2.38 percent after 12 months and from 7.42 ± 7.77 percent to 2.47 ± 2.50 percent in the Posiforlid group. Although significantly lower, there was no significant difference between the two studied groups. No statistically significant changes were registered in the studied quantitative parameters. Using the EFT test, great improvements were registered regarding symptomatology, with a score increasing from 29.99 ± 8.60 to 39.10 ± 5.08 in the control group and from 27.35 ± 9.24 to 38.35 ± 4.62 in the other group. Again, the same statistical result was registered on this variable.

**Conclusions:** The improvement of tear film stability, ocular surface inflammatory condition, and subjective symptoms during IPL therapy sessions and the first year of observation after the completion of the treatment was not necessarily increased by the additional use of a heated eye mask.

**Abbreviations:** IPL = intense pulsed light therapy, EFT = eye fitness test, NIFBUT = non-invasive first break-up time, NIABUT = non-invasive average break-up time, OSIE = ocular surface inflammatory evaluation, TFSE = tear film stability evaluation, CTMH = central tear meniscus height, TTMH = thinnest tear meniscus height, DED = dry eye disease, MGD = meibomian gland dysfunction, SD = standard deviation

## Introduction

Dry eye disease (DED) is a multifactorial disease of the ocular surface characterized by tear film instability or tear film deficiency, accompanied by various symptoms that have a strong everyday life impact [**1**,**2**]. Dry eye disease represents a result of multiple contributing factors, in different combinations, proving the unique dynamic of the disease in every person [**3**]. Contributing factors include environmental conditions, sociodemographic elements, and personal aspects such as systemic diseases, medication, surgeries, or local ocular factors [**4**].

Understanding that DED is not only the insufficiency of the aqueous part of the tear film but also excessive tear evaporation that can lead to chronic inflammation of the ocular surface together with meibomian gland dysfunction has been the key point for new therapies and ways of approaching this pathology [**5**]. Traditional strategies have failed the test of time, proving ineffective and underlining the need for exploring new horizons in treating MGD [**6**].

Intense pulsed light therapy is one of the novel therapies in treating dry eye disease caused by MGD, used worldwide for its simplicity and great effect, highly demonstrated during the past 10 years [**7**]. Most treatment schemes include 4 sessions, spaced 2 weeks and 4 weeks apart, the effect being cumulative [**8**]. This therapy goes to the root of the MGD, by treating the abnormalities in the meibomian gland ducts and adjusting their functions [**9**].

This study investigated the tear film stability changes and ocular surface health in patients undergoing combined therapy (IPL treatment and thermal mask) compared with patients undergoing IPL treatment alone. Through this study, we observed and compared the objective and subjective efficiency when applying IPL sessions, and during the first year after treatment, considering the two study groups mentioned above. 

## Materials and methods

*Study design and population*

This was an open-label, retrospective monocentric, and interventional study, with the consecutive enrolments of the patients already diagnosed with dry eye disease from May 2021 until May 2023. Objective assessments and subjective points of view were performed with IPL therapy sessions, on day 1, two weeks from the first (Session 2), and one month from the previous (Session 3 and Session 4). Also, the same measurements were made after 3 months, 6 months, and 1 year from completion of the IPL treatment. Study group 1 represented the control group, in which patients completed the IPL treatment alone and study group 2 represented patients who completed IPL treatment and used a Posiforlid thermal mask every day, from the beginning of the IPL treatment until completion, with a 1-year follow-up period after IPL treatment. 

*Inclusion and exclusion criteria*

110 patients were included in the study, meaning 220 eyes were studied. All patients completed the 4 session IPL treatment as follows: day 1, day 15, day 45, and day 75 with 3 follow-ups at 3, 6, and 12 months after IPL treatment completion. Study group 1 (Control group) included 73 patients (146 eyes), meanwhile, study group 2 included 37 patients encompassing 74 eyes. No changes in topical treatment have been made during treatment and follow-up. No ocular surgeries have been performed. No other pathological changes have been registered.

Selection criteria were in strict accordance with the guidelines from the International Workshop on MGD [**10**], mostly for patients over 18 years old, with a positive diagnosis of dry eye disease due to MGD, already under treatment with artificial tears for at least 3 months. Criteria for excluding the patients were: systemic pathology with a known impact on tear film stability, ocular surgery during the past 3 months, other ocular inflammatory conditions during the past 6 months (uveitis, keratitis, episcleritis), glaucoma, skin pathology (pigmentation, trauma, cancer), patients wearing contact lenses.

*Ocular surface evaluation*

An objective examination of the tear film used a tear check imaging device (ESW vision, Houdan, France), completed by Sirius Scheimpflug Camera.

CTMH and TTMH measuring was made after fluorescein dye instillation into the eye, followed by high-resolution image capturing, which reflected the presence and measured the quantity of the tear meniscus. These measurements analyzed the quantitative parameters of the tear film.

OSIE is a measurement performed 120 seconds after fluorescein dye instillation, which should normally be eliminated from the ocular surface by that time, but in inflammatory conditions is found for a longer time on the ocular surface, by adhering to the alterations of the cornea and conjunctiva caused by inflammation. 

TFSE highlights the number and intensity of micro-deformations appearing on the ocular surface due to unstable tear film, assessed during a 10-second examination, with an automatically calculated score, ranging from 18 to 1800. 

Tear film break-up time was objectively determined using a Sirius Scheimpflug Camera, with two determinations: non-invasive first break-up time (NIFBUT) and non-invasive average break-up time (NIABUT). The patient was asked to blink twice and afterward keep the eyes open as long as possible, meanwhile, the device captured multiple high-resolution images to detect the first break in the tear film and the average time for the following disruptions. NIFBUT values above 15s and NIABUT values above 10s were linked to normal values.

Subjective evaluation was performed by using an Eye Fitness Test (EFT) made up of four questions regarding general well-being with yes/no answers, nine questions regarding symptoms and analysis of eye fitness with never/sometimes/regularly/often and always answers, graded descending from 4 to 0 and two questions graded the same, concerning environmental influences. Therefore, higher scores in subjective evaluation were linked to better results.

*Statistical analysis*

In the present study, we used SPSS Statistics software, version 29.0, by IBM Corporation in Armonk, NY, USA, for data analysis. Using the GRANMO calculator, version 7.12 we determined the study sample size. With an accepted alpha risk of 0.05 and a beta risk of 0.2, 140 eyes were necessary to obtain a significant mean difference. An anticipated 10% dropout rate and a standard deviation of 3.8 were considered, an aspect already considered in previous works [**11**].

Differences between the first and last visit were noted as Δ = Last Visit - Baseline. For comparisons within the group, Student’s T-tests or Wilcoxon signed-rank tests were used, while for comparisons between groups, Student’s T-test or Mann-Whitney U test. A level of P < 0.05 was considered significant for all comparisons.

## Results

**Table 1:** Dry eye disease changes between IPL alone and the use of masks during sessions

Variables	Session time	Thermal mask group	Control group	*P value a*
Subjective Symptoms				
EFT, score points, mean ± SD [range]	Time 1	27.35 ± 9.24 [4 to 43]	29.99 ± 8.60 [11 to 43]	0.28
	Time 2	34.11 ± 9.56 [10 to 44]	35.20 ± 7.72 [12 to 44]	0.77
	Time 3	33.62 ± 8.66 [19 to 44]	37.07 ± 5.77 [19 to 44]	0.28
Tear Film Stability				
NIFBUT, seconds, mean ± SD [range]	Time 1	9.96 ± 6.48 [1.20 to 17.00]	9.07 ± 5.80 [1.10 to 17.00]	0.44
	Time 2	11.07 ± 5.72 [2.60 to 17.00]	11.32 ± 5.57 [1.20 to 17.00]	0.78
	Time 3	10.35 ± 5.95 [1.40 to 17.00]	11.01 ± 5.78 [1.10 to 17.00]	0.50
NIABUT, seconds, mean ± SD [range]	Time 1	11.77 ± 5.08 [3.10 to 17.00]	10.72 ± 4.90 [2.30 to 17.00]	0.39
	Time 2	12.56 ± 4.42 [1.30 to 17.00]	12.39 ± 4.70 [2.80 to 17.00]	0.69
	Time 3	12.10 ± 4.69 [3.10 to 17.00]	12.47 ± 4.66 [2.20 to 17.00]	0.92
TFSE, score points, mean ± SD [range]	Time 1	391.11 ± 456.457 [18 to 1800]	310.56 ± 389.54 [10 to 1800]	0.44
	Time 2	206.30 ± 237.85 [18 to 935]	261.51 ± 353.20 [18 to 1747]	0.73
	Time 3	213.27 ± 247.49 [18 to 1325]	202.32 ± 237.55 [18 to 1305]	0.92
Tear Film Quantity				
CTMH, mm, mean ± SD [range]	Time 1	0.47 ± 0.22 [0.13 to 1.04]	0.43 ± 0.21 [0.09 to 1.04]	0.50
	Time 2	0.43 ± 0.19 [0.13 to 0.87]	0.44 ± 0.20 [0.11 to 1.05]	0.46
	Time 3	0.46 ± 0.19 [0.17 to 1.02]	0.43 ± 0.18 [0.14 to 0.97]	0.63
TTMH, mm, mean ± SD [range]	Time 1	0.59 ± 0.29 [0.16 to 1.42]	0.52 ± 0.27 [0.17 to 1.55]	0.18
	Time 2	0.51 ± 0.23 [0.20 to 0.99]	0.52 ± 0.26 [0.18 to 1.69]	0.98
	Time 3	0.53 ± 0.24 [0.21 to 1.13]	0.50 ± 0.22 [0.15 to 1.47]	0.44
Surface Evaluation				
OSIE Type 1, percentage, mean ± SD [range]	Time 1	7.42 ± 7.77 [0 to 35]	7.18 ± 7.93 [0 to 53]	0.87
	Time 2	4.59 ± 5.03 [0 to 21]	6.77 ± 6.68 [0 to 45]	0.06
	Time 3	4.32 ± 4.26 [0 to 19]	5.43 ± 4.98 [0 to 24]	0.51
OSIE Capture time, seconds, mean ± SD [range]	Time 1	130.77 ± 10.63 [107 to 150]	130.17 ± 10.22 [112 to 149]	0.66
	Time 2	132.76 ± 10.98 [112 to 150]	129.04 ± 9.31 [112 to 153]	0.16
	Time 3	129.18 ± 9.64 [110 to 149]	129.13 ± 8.57 [113 to 150]	0.93
CTMH = Central tear meniscus height (below iris), DED = Dry eye disease, EFT = Eye Fitness Test, NIABUT = Non-invasive average break up time, NIFBUT = Non-invasive first break up time, OSIE = Type 1 Ocular surface inflammatory evaluation (with fluorescein sodium and oxybuprocaine hydrochloride), SD = Standard deviation, TFSE = Tear film surface evaluation, TTMH = Thinnest tear meniscus height. a U of Mann Whitney.				

The study included 110 participants, 73 patients in the control group, undergoing IPL treatment alone, and 37 patients in the thermal mask group, undergoing IPL treatment alongside thermal mask usage. All the participants in the study registered an average age of 51.88 (SD = 15.26), from 18 years old to 86 years old, of whom 69 were female participants representing 62.7%, and 41 were male participants representing 37.3%. The study results are systematically organized in the results tables. 

**Table 1** specifically shows differences between IPL sessions regarding symptomatology and tear film characteristics comparing both study groups.

On the other hand, **Table 2** shows both study groups during follow-up evaluations (at 3, 6, and 12 months), considering the same variables.

**Table 2:** Dry eye disease changes between IPL follow-up and the use of a mask

Variables	Follow-up	Thermal mask group	Control group	*P value a*
Subjective Symptoms				
EFT, score points, mean ± SD [range]	3 months	35.64 ± 7.56 [20 to 43]	38.41 ± 4.27 [24 to 44]	0.85
	6 months	35.44 ± 6.23 [23 to 44]	37.68 ± 5.99 [14 to 44]	0.88
	12 months	38.35 ± 4.62 [23 to 44]	39.10 ± 5.08 [19 to 44]	0.28
Tear Film Stability				
NIFBUT, seconds, mean ± SD [range]	3 months	12.33 ± 5.32 [2.40 to 17.00]	11.36 ± 5.49 [1.10 to 17.00]	0.17
	6 months	11.99 ± 5.26 [2.50 to 17.00]	12.29 ± 5.01 [1.30 to 17.00]	0.99
	12 months	15.13 ± 3.44 [3.80 to 17.00]	13.82 ± 4.94 [1.10 to 17.00]	0.50
NIABUT, seconds, mean ± SD [range]	3 months	13.48 ± 3.94 [5.60 to 17.00]	12.81 ± 4.22 [3.10 to 17.00]	0.23
	6 months	13.61 ± 3.85 [4.20 to 17.00]	13.45 ± 3.93 [3.20 to 17.00]	0.99
	12 months	15.84 ± 2.26 [7.80 to 17.00]	14.79 ± 3.72 [1.10 to 17.00]	0.92
TFSE, score points, mean ± SD [range]	3 months	165.94 ± 200.61 [18 to 748]	168.51 ± 210.382 [18 to 1410]	0.41
	6 months	164.71 ± 164.62 [18 to 923]	163.54 ± 157.89 [18 to 653]	0.84
	12 months	97.38 ± 105.98 [18 to 645]	114.40 ± 122.90 [18 to 640]	0.56
Tear Film Quantity				
CTMH, mm, mean ± SD [range]	3 months	0.41 ± 0.14 [0.21 to 0.75]	0.39 ± 0.13 [0.11 to 0.85]	0.91
	6 months	0.41 ± 0.13 [0.24 to 0.89]	0.38 ± 0.12 [0.18 to 0.82]	0.62
	12 months	0.39 ± 0.14 [0.21 to 1.06]	0.38 ± 0.13 [0.16 to 0.92]	0.77
TTMH, mm, mean ± SD [range]	3 months	0.48 ± 0.18 [0.22 to 0.94]	0.45 ± 0.19 [0.16 to 1.55]	0.55
	6 months	0.45 ± 0.14 [0.28 to 0.92]	0.43 ± 0.14 [0.21 to 0.97]	0.77
	12 months	0.42 ± 0.15 [0.24 to 1.06]	0.41 ± 0.14 [0.16 to 0.98]	0.84
Surface Evaluation				
OSIE Type 1, percentage, mean ± SD [range]	3 months	3.92 ± 3.49 [0 to 16]	3.83 ± 3.87 [0 to 31]	0.98
	6 months	4.59 ± 4.33 [0 to 19]	3.55 ± 3.06 [0 to 18]	0.50
	12 months	2.47 ± 2.50 [0 to 9]	2.24 ± 2.38 [0 to 16]	0.91
OSIE Capture time, seconds, mean ± SD [range]	3 months	128.46 ± 8.81 [114 to 149]	127.70 ± 7.47 [116 to 149]	0.88
	6 months	127.12 ± 7.06 [113 to 149]	128.49 ± 7.94 [113 to 149]	0.90
	12 months	126.46 ± 6.37 [118 to 146]	127.42 ± 6.94 [113 to 148]	0.15
CTMH = Central tear meniscus height (below iris), DED = Dry eye disease, EFT = Eye Fitness Test, NIABUT = Non-invasive average break up time, NIFBUT = Non-invasive first break up time, OSIE = Type 1 Ocular surface inflammatory evaluation (with fluorescein sodium and oxybuprocaine hydrochloride), SD = Standard deviation, TFSE = Tear film surface evaluation, TTMH = Thinnest tear meniscus height. a U of Mann Whitney.				

**Fig. 1:**
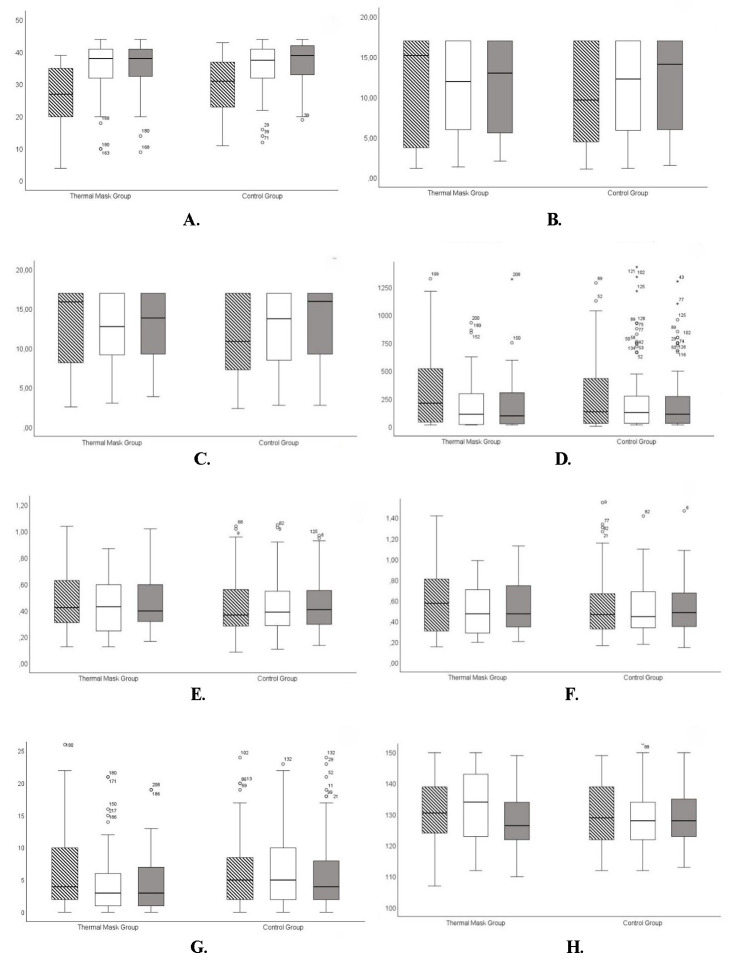
The striped pattern represents the Time 1 evaluation, the white pattern represents the Time 2 evaluation, and the grey pattern represents the Time 3 evaluation

**Fig. 2:**
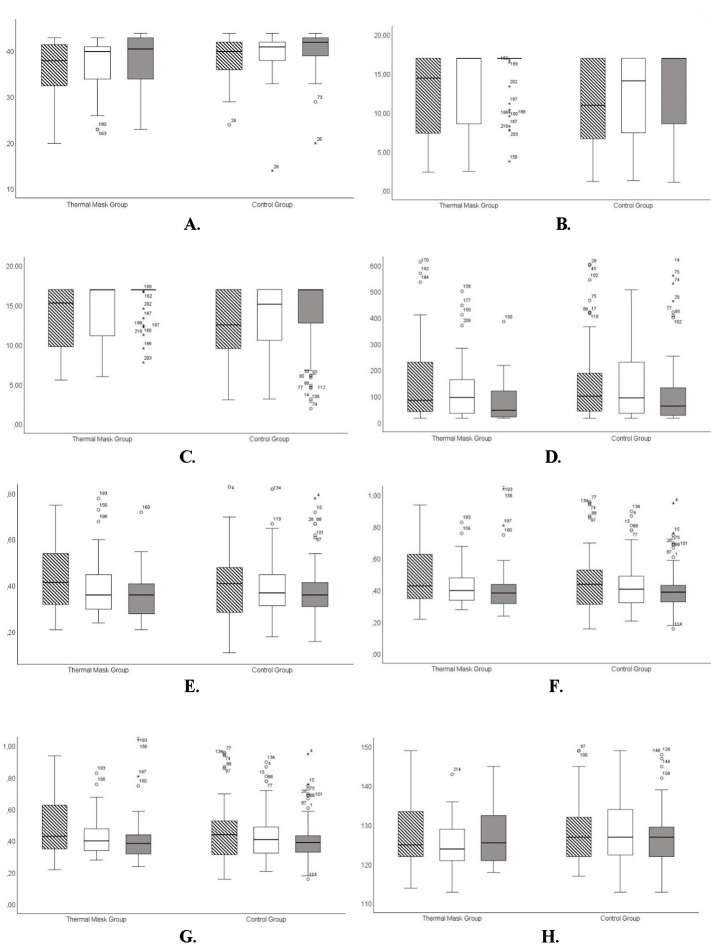
The striped pattern represents 3 months follow-up, the white pattern represents 6 months follow-up, and the grey pattern represents 12 months follow-up

**Fig. 1** presents a detailed analysis and integrates eight box & whisker plots comparing results between sessions and separated by thermal mask group and control group, each of them underlining important elements such as **A.** EFT scores, **B.** NIFBUT, **C.** NIABUT, **D.** TFSE scores, **E.** CTMH, **F.** TTMH, **G.** OSIE, and **H.** OSIE capture time. **Fig. 2** highlights the same variables between the thermal mask and the control groups in the follow-up after IPL treatment.

Significant differences regarding subjective symptoms and tear film qualitative assessment and stability in time were demonstrated in this study. Moreover, during the IPL treatment sessions and the follow-up period, less important differences in quantitative assessment parameters were highlighted. Nevertheless, no statistically significant differences were noted between the two study groups, IPL alone or IPL with thermal mask use, no matter the studied variables. With no exception, improvement reflected in studied parameters between and after IPL multi-session treatment was not significantly increased when combined with thermal mask use. 

## Discussion

Our study first fortified the benefit of the IPL treatment in improving the tear film stability symptomatology, and efficiency, which proved its stability in time, aspects already described in previous studies like Toyos et al. [**12**] or Qin et al. [**13**]. The question regarding the long-time efficiency of the additional Posiforlid heated eye-mask therapy was also raised. 

When considering the patient’s quality of life, we concluded that a non-statistically significant result between the two study groups that was demonstrated in the study might influence patients to rather opt for a 4-session IPL therapy with a single session repetition every 3-6 months, than the everyday use of a heated eye mask. The first option is less time-consuming than the second one, although more expensive. 

Liangzhe Li et al. [**14**] underlined in their study that the study group combining IPL treatment with a heated eye mask registered much better results concerning tear film stability and inflammatory response, than the study group under IPL treatment alone. Still, measurements in their study were made only during the IPL treatment, the last ones on day 84 of treatment, and after 3 sessions of IPL therapy, compared to our study that included 4 IPL sessions (as recommended by the manufacturer) and a much longer period of observation and assessment. In their study, the mean age for the IPL group was 29.88 years and for the IPL and heated eye mask group was 28.06, while in our study the mean age for the IPL group was 54.31 years and for the IPL and heated eye mask group was 47.08, with a much wider range of age compared to Liangzhe Li et al. study. These two differences in the design of the studies could be a source of diminishing the effect of the heated eye mask use. 

Moreover, the study of Ling Xu et al. [**15**] showed the efficiency of the heated eye mask as a single therapy and IPL therapy for treating patients who systematically wear contact lenses. In their study, they underlined that the IPL proved more efficient in improving the stability of the tear film in the long time run, compared with a heated eye mask, which proved to be efficient for a very short period. This might come as an additional argument for the result of our study, proving that under the effect of IPL therapy, using a heated eye mask is not that efficient. 

Nevertheless, our study aligned with Trone MC et al. [**16**] study regarding quantitative measurements of the tear film, such as CTMH or TTMH, which remained unmodified irrespective of the therapy applied. These findings showed that therapies for meibomian gland dysfunction target the improvement of the meibomian glands’ functionality, tear film stability, and quality, and not necessarily the quantity of the tear film. 

Although it was meant to come up with answers to critical questions raised in previous studies, our study also had limitations. First, it did not include many patients treated only with a thermal eye mask to compare the effect of the thermal eye mask alone. Presenting the response of the IPL alone, a heated eye mask was considered only as an additional therapy for IPL treatment in this study. Furthermore, it did not include an evolutive comparison of the meibography assessment. Also, the use of a non-standardized questionnaire in evaluating the severity of symptoms, decreased the relevance of a potential comparison between studies, even though the design of the EFT was very similar to other validated tests, but graded differently. Nevertheless, the need for a standardized point of view considering multiple variables such as age, grade of severity, risk factors, symptomatology, patient satisfaction, patient expectations, and others when deciding on a specific dry eye therapy management should be considered in future studies.

## Conclusion

The improvement of tear film stability, ocular surface inflammatory condition, and subjective symptoms during IPL therapy sessions and the first year of observation after the completion of the treatment is not necessarily increased by the additional use of a heated eye mask. These findings encourage using IPL therapy, because of its results and small number of sessions during a longer period. 

**Conflict of Interest**

The authors state no conflict of interest.

**Informed Consent and Human and Animal Rights Statement**

Informed consent has been explained, available, and gathered from all patients who participated in the study.

**Authorization for the use of human subjects**

Ethical approval: This study was approved by the Ethics Committee of “Victor Babeş” University of Medicine and Pharmacy, Timişoara, Romania, record number 48/2021. Also, it is in line with the principles of the Helsinki Declaration, institutional guidelines, and national principles. 

**Acknowledgments**

None.

**Sources of Funding**

None.

**Disclosures**

None.
